# Biomechanics of Additively Manufactured Metallic Scaffolds—A Review

**DOI:** 10.3390/ma14226833

**Published:** 2021-11-12

**Authors:** Karim Elhattab, Mohamed Samir Hefzy, Zachary Hanf, Bailey Crosby, Alexander Enders, Tim Smiczek, Meysam Haghshenas, Ahmadreza Jahadakbar, Mohammad Elahinia

**Affiliations:** Department of Mechanical, Industrial & Manufacturing Engineering, College of Engineering, The University of Toledo, Toledo, OH 43606, USA; karim.elhattab@utoledo.edu (K.E.); zachary.hanf@rockets.utoledo.edu (Z.H.); Bailey.crosby@rockets.utoledo.edu (B.C.); alexander.enders@rockets.utoledo.edu (A.E.); Tim.Smiczek@rockets.utoledo.edu (T.S.); Meysam.haghshenas@utoledo.edu (M.H.); ar.jahadakbar@gmail.com (A.J.); mohammad.elahinia@utoledo.edu (M.E.)

**Keywords:** 3D printing, additive manufacturing, biomechanics, metallic scaffolds, titanium alloy Ti6Al4V

## Abstract

This review paper is related to the biomechanics of additively manufactured (AM) metallic scaffolds, in particular titanium alloy Ti6Al4V scaffolds. This is because Ti6Al4V has been identified as an ideal candidate for AM metallic scaffolds. The factors that affect the scaffold technology are the design, the material used to build the scaffold, and the fabrication process. This review paper includes thus a discussion on the design of Ti6A4V scaffolds in relation to how their behavior is affected by their cell shapes and porosities. This is followed by a discussion on the post treatment and mechanical characterization including in-vitro and in-vivo biomechanical studies. A review and discussion are also presented on the ongoing efforts to develop predictive tools to derive the relationships between structure, processing, properties and performance of powder-bed additive manufacturing of metals. This is a challenge when developing process computational models because the problem involves multi-physics and is of multi-scale in nature. Advantages, limitations, and future trends in AM scaffolds are finally discussed. AM is considered at the forefront of Industry 4.0, the fourth industrial revolution. The market of scaffold technology will continue to boom because of the high demand for human tissue repair.

## 1. Introduction

Do you remember the “Six Million Dollar Man”, an American science fiction and action television series created in the seventies? Maybe this show inspired researchers to look into developing artificial and bio organs and bionic implants. Could you imagine having an artificial nose with an integrated bio sensing system [1]? What about a bionic ear [2]? What about a bionic eye [3]? These bio organs are no longer available, only in fiction stories or shows, but they are being created as prototypes for further developments. Professor Michael C. Alpine from the University of Minnesota received in 2017 the Presidential Early Career Award for Scientists and Engineers for his work on designing and building a bionic eye that can image projections by printing layers of photosensitive material in a hemispherical shape that detected light [3]. Professor M.C. Alpine also developed a working bionic ear at Princeton University. It was printed using cartilage cells in a hydrogel matrix, structural silicon, and silicon infused with silver nanoparticles [2]. A bioelectronics nose that can be used for the detection of Salmonella contamination using Odorant Binding Protein-derived peptide and carbon nanotube field effect transistor was developed by a group of researchers in Korea [4]. A proof of concept for an odor-perceptive artificial nose composed of a biocompatible sensing platform was proposed by a group of researchers at the Stevens Institute of Technology [5].

The market size of artificial organs and bionic implants in 2019 was USD 25.9 billion [6]. According to a recent market analysis [7], the global market size of these implants is expected to grow at a compound annual growth rate of 10.2% from 2019 to 2026. Bionic implants and artificial organs are constructed and 3D printed with the goal to create bionic tissues and implants. Tissue engineering was defined as “the development of bio-substitutes that can be utilized to restore, maintain, or improve tissues damaged or lost by various disease conditions” [8]. Autograft is a tissue or organ that is transplanted from one part to another part of the same body. Using autografts is the traditional way that has been used for tissue regeneration. However, this method has its limitations including the availability of donor tissues [9]. Scaffolds are currently used in tissue engineering to repair tissues and organs. Cells are collected and then cultured in a platform, namely, a scaffold. Scaffolds are used as a support structure for cell growth, thus helping cell generation [10]. Several conventional techniques have been identified using polymers and textiles to fabricate scaffolds including freeze drying and gas foaming, among others [11]. However, these techniques not only are time consuming and labor intensive, but they also cannot control the pore characteristics of the scaffolds, including their size and distribution. During the past 20 years, additive manufacturing (AM) has emerged as the method of choice to print prototypes for different industries and applications including scaffolds for the medical industry. In AM, a part is printed layer-by-layer, and several AM methods have been developed using different processes and materials. The factors that affect the scaffold technology are the design of the scaffold, the material used to build the scaffold, and the fabrication process of the scaffold [12].

## 2. Scaffolds

### 2.1. What Are They?

It is necessary to understand the mechanical properties and the chemical composition of the tissues that scaffolds are used to restore their forms and functions. Bones, cartilage, and tendons are considered connective tissues. They provide support to the body and have a lower cellular content than other tissue types. Bone is the strongest material in the body with a strength up to 300 MPa [13]. Cartilage on the other hand is a viscoelastic material [14].

Tissues can be considered as complex composites of natural biopolymers, inorganic component, and cells. Synthetic materials do not reproduce the complexity of natural tissues, which does not allow for good tissue integration [15,16,17,18]. Also using natural materials are associated with immune response concern [19] if they are not properly decellularized [20].

The extracellular matrix (ECM) forms the foundation of tissues. The major structural component of native tissues is proteins, collagen, and filamentous protein fibrils protect the tissues and their cells. Collagen has been used in scaffolds to regenerate tissues. ECM is difficult to recreate with synthetic materials because it has a complex and organized three-dimensional structure. Decellularized native tissues have thus been used successfully in tissue engineering.

Scaffolds interact with cells allowing tissue regeneration. The receptors on the surface of the cells interact with the extracellular environment including its proteins [21]. It is thus important to determine the effects of scaffolds materials on cell phenotype. Cells phenotype vary when cultured in different geometrical environments. It is thus important to understand the influence of scaffold geometry across multiple length scales on cell phenotype.

Success in tissue engineering requires developing products that are patient specific. Recently, scaffolds have been generated using decellularized organs that retain ECM structure while employing patient’s own cells. Whole organs have been generated accordingly including heart, lung, liver, skin, and kidney [22,23,24,25,26,27,28]. However, several factors need to be considered including availability of donor organs before these scaffolds can used for organ transplant. Also, the life time of these organs is not known [29]. Nevertheless, this work highlights the important role of the ECM.

The fabrication of a scaffold encompasses microscale and macroscale levels. At the microscale level, the scaffold allows for cells survival and function. At the macroscale level, the scaffold provides adequate support during tissue repair. In what follows we will briefly review the conventional methods to fabricate scaffolds. In these methods a porous polymer structure is constructed. However, it is difficult to replicate the complex native structures using the conventional methods [30]. Recent research indicates that AM has a great potential to produce functional scaffolds and organs [31].

### 2.2. Conventional Methods to Fabricate Scaffolds

**Figure 1:**
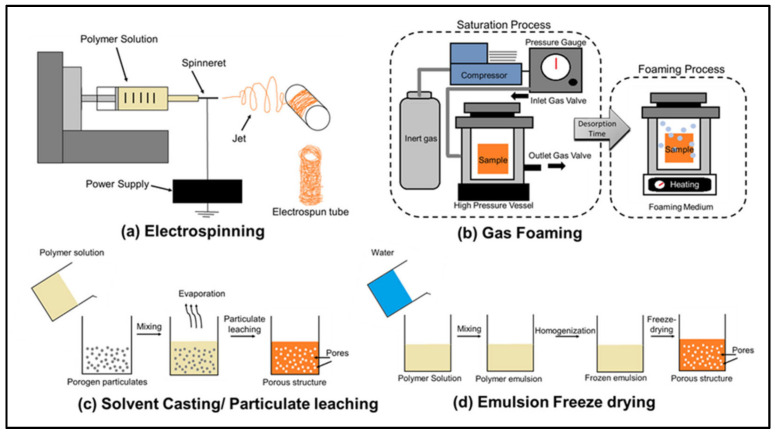
Four conventional scaffold fabrication methods: (**a**) electrospinning; (**b**) gas foaming; (**c**) solvent casting/particulate leaching; (**d**) freeze drying [32].

Most of the conventional methods listed below are used to fabricate polymer scaffolds. Figure 1 includes a schematic showing four of these conventional methods [32]. Also, Table 1 summarizes the advantages and disadvantages of each of these methods [11].

#### 2.2.1. Solvent Casting and Particle Leaching

This is an easy and low cost technique where a polymer is dissolved in an organic solvent. Salt particles are then added to the solution. The mixture is then cast in a three-dimensional mold to produce the scaffold. The solvent evaporates to create a structure made of the particles and the polymer. This structure is then placed in a bath where the particles are dissolved and the final structure is a porous one [33]. Scaffolds obtained using this method can have a high porosity up to 90% [34]. This technique uses only thin membranes and hence is time consuming [35]. Furthermore, toxic solvents are used which can also take a lot of time to evaporate.

#### 2.2.2. Freeze Drying

Freeze drying, also known as lyophilisation or cryodesiccation [36] is a low-temperature dehydration process that involves freezing the product then removing ice by sublimation, which is the transition of a substance directly from the solid to the gas state, without passing through the liquid state. A synthetic polymer is dissolved and the solution is cooled under the freezing point. The solid solvent is then evaporated by sublimation creating a porous scaffold [37]. The pores correspond to the volume occupied by the ice which can be controlled allowing thus to manage the pore sizes [11]. This method is widely used in the fabrication of scaffolds because of using ice instead of an organic solvent [38]. For example, Min and Lee [39] fabricated a 3-D scaffold using chitosan nanoparticles. However this technique has many disadvantages including using cytotoxic solvents to mix the polymer and the generation of small and irregular pore sizes in the range of 15 to 35 μm [40].

#### 2.2.3. Thermal Induced Phase Separation

Thermally Induced Phase Separation (TIPS) is a process in which the membrane formation is induced by cooling the polymer solution [41]. The TIPS process is applied to polymers with poor solubility and used only for thermoplastics and is used to fabricate thermoplastic crystalline polymer scaffolds. In the first stage the polymer is molten and fed into an extruder. The molten polymer is mixed with a solvent/non-solvent mixture that is selected so that the polymer is dissolved only above a certain temperature creating a homogenous solution. A flat sheet is formed by extruding the hot solution through a slit die, while a hollow-fiber is formed by extruding the hot solution though a spinneret. The solution is then cooled down below the dissolving temperature and a polymer rich and solvent-rich phases are formed [34]. The polymer rich phase is allowed then to solidify. The solvent-rich phase is embedded in the rigid polymer matrix and is extracted with liquids. This microporous polymer matrix is then dried resulting in a construct with a relatively porous, nanoscale fibrous network.

#### 2.2.4. Gas Foaming

Gas foaming is a scaffold fabrication method that avoids using solvents by generating gas bubbles within a polymer. The polymer is first molded at high temperature. It is then pressurized with carbon dioxide where the gas infiltrates the polymer creating pores for tissue in growth. It has been reported that gas foaming produces structures with a pore size of 30 to 700 µm and porosity up to 85% [42]. Solvents are not used in gas foaming, which represents an advantage of this method as there is no solvent in the fabricated scaffold, making it possible to incorporate sensitive bioactive molecules.

#### 2.2.5. Electrospinning

Electrospinning technique is used to fabricate nanofibers of polymers, metals and ceramics where the diameters of the fibers can be hundreds of nanometers [43]. Electrospinning includes a high voltage power supply, grounded collector, and positively charged capillary tube packed with polymer fluid. The high voltage electric field is applied to form fibers from polymer fluid that is delivered through the capillary tube. A liquid polymer jet is formed and deposited on the collector. The jet is then solidified to form the scaffold [34,42]. While complicated, this technique has proven reliable in developing nanofibrous scaffolds. Nanoparticles can be mixed with polymers and electrospun to produce the scaffolds. Solvents are used to separate the nanoparticles and dissolve the polymer. Many biopolymers such as collagen and chitosan have been successfully electrospun to produce scaffolds for tendons [44]. The major concern is using organic solvents in the process.

## 3. Design of Scaffolds

Each tissue has its own requirement for scaffold design. Different tissue types have different composition, density, nanostructure, and microstructure of extracellular matrix (ECM). In designing a scaffold for tissue engineering, it is critical to understand the material properties and the chemical composition of native tissue. Connective tissue, which includes bones, cartilage, and tendons, provide structural support to the body and tends to have a low cellular content relative to other types of tissues.

Properties of scaffolds, degradation and biocompatibility are considered in scaffold design. The tissue to be augmented or replaced dictates the form and function of scaffolds that must be designed keeping in mind the chemical and material properties of the native tissue as well as the cell interactions, Progress has been seen in organs such as skin, bladder, cornea, trachea, and blood vessels. Refs [45,46,47,48,49,50] as well as in connective tissues, such as bone and cartilage [35], in addition to nervous tissue [51] and muscle. On the other hand, organs such as liver, kidney, heart, and pancreas, still present a significant challenge at present [52].

The design of the scaffolds as mentioned earlier directly modulate the stiffness of the fabricated parts and affect the level of bone ingrowth. AM Ti-6Al-4V cellular structures are widely used in the biomedical field. Several studies have focused on understanding the different design parameters on AM Ti-6Al-4V cellular structures. Design parameters include cell shape, porosity and pore sizes which will be discussed in what follows.

### 3.1. Cell Shape

**Figure 2:**
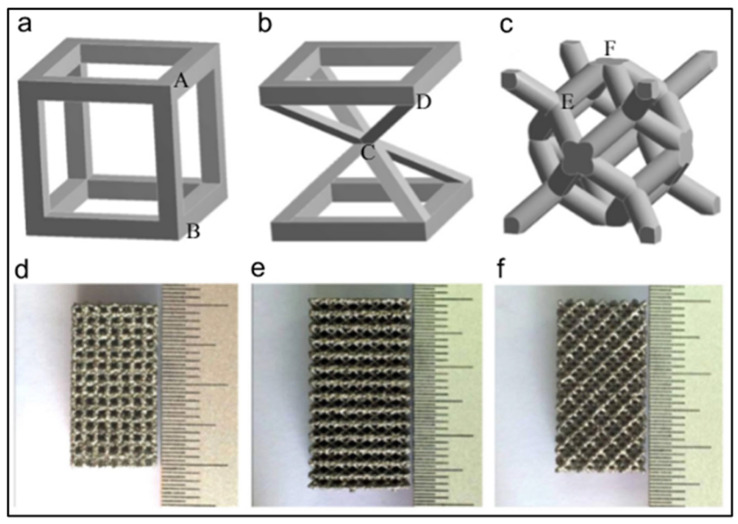
Cubic, G7, and rhombic dodecahedron element in the materialize software (**a**–**c**) and the corresponding Ti-6Al-4Vprototype blocks fabricated by EBM method (**d**–**f**) [53].

The cell shape of the unit cell highly affects the mechanical properties of open cellular structures [53]. Zhao, et al. [53] and Li, et al. [54] conducted two studies to determine the influence of three cell shapes on the mechanical properties of Ti-6Al-4V mesh arrays fabricated by electron beam melting. The cells were the cubic, G7 and rhombic dodeca-hedron cells. Figure 2 shows the three cells and the corresponding Ti-6Al-4V prototype blocks.

Li, et al. [54] used Materialize software to fabricate the prototypes. A constant strut thickness of 0.5 mm was used to build the units allowing for different cell sizes, different densities, and different porosities. They reported that for the same densities, the cubic mesh possesses the highest modulus, while the G7 mesh has the lowest. They also reported that equivalent Young’s modulus increases linearly from 0.5 to 15 GPA with the increase in density for three types of meshes. This is comparable with those of trabecular (0.05–3 GPa) and cortical bone tissue (10–25 GPa) [54]. The compressive strength varied between 10 and 300 MPa and was found also to vary linearly with the density for the meshes with same cell shape. They also reported that the cubic and rhombic dodecahedron meshes exhibit brittle deformation behavior, while meshes with G7 structure perform as ductile metallic foams. They concluded that optimizing the cellular structures fabricated by the EBM can improve their brittle deformations.

Zhao, et al. [53] also reported that Young’s modulus and strength decreased in the order of cubic, rhombic dodecahedron, and G7 structure. For instance, Young’s moduli of the rhombic dodecahedron and G7 structures were less than that of the cubic by 57.72% and 83.89%, respectively. Also, the strength of the rhombic dodecahedron and G7 structures were less than that of the cubic by 42.86% and 68.88% respectively. Zhao, et al. [53] also found that the ratcheting rate decreased in the order of G7, rhombic dodecahedron and cubic meshes. Cyclic ratcheting is a measure of increasing accumulation of strain in meshes due to the strut bending during cyclic fatigue. Zhao, et al., further reported that in low and high cycle fatigue region, the fatigue damage strain of cubic mesh was very small. On the other hand, the fatigue damage increased gradually, in particular in the low cycle fatigue for the G7 and rhombic dodecahedron meshes. They reported that the fatigue strength (S-N diagram) is affected by the shape of the cells of the mesh and was the highest for the cubic mesh and lowest for the G7 mesh, which is consistent with their ratcheting rates results. They concluded that cell shapes of Ti-6Al-4V affect the fatigue strength of the corresponding cellular solids fabricated by the EBM technique.

### 3.2. Porosity and Pore Size

Wang, et al. [55] reported that the mechanical and biological properties of cellular materials are affected greatly by their porosity, pore interconnectivity and pore size. Porous metals with predefined external shape and complex internal architecture can be fabricated using AM technologies, and in particular SLM and EBM. The typical design process of porous metallic implants includes (1) design of scaffold, (2) AM, and (3) post processing, which includes heat-treatment and surface modification. Bone scaffolds need to be highly porous with an interconnected pore network to allow bone ingrowth and must have mechanical properties that reduce stress shielding. Higher porosity allows for more bone ingrowth. In another study, Ti-6Al-4V specimens with controlled porosity have been designed and tested experimentally to evaluate their Young’s modulus and yield stress [56]. Structures with densities of 20%, 42%, and 60% and a cell size of 0.83 mm were manufactured using tetrahedral unit cells. Young’s modulus and yield stress increased with the increase of the density. Murr, et al. [57] conducted a study on Ti-6Al4V implants and demonstrated that when the porosity changed from 59% to 88%, the elastic modulus decreased from 3.03 to 0.58 GPA [57]. In another study, Pattanyak, et al. [58] have shown that when the porosity changed from 55% to 75%, the compressive strength decreased from 120 MPa to 35 MPa [58]. So, while increasing the porosity improves bone ingrowth, it does decrease the stiffness and strength.

Wang, et al. [55] report that there is a controversy in the literature about the optimal pore size and its influence on bone ingrowth. Larger surface area is associated with smaller pores. One can thus argue that scaffolds with smaller pore size have more space for bone ingrowth. Yet, Taniguchi, et al. [59] found that 600 µm and 900 μm Ti porous scaffolds had higher bone ingrowth than 300 µm Ti porous scaffolds when implanted into rabbit tibia.

## 4. Processes to Fabricate AM Scaffolds

Bioprinting a tissue consists of assembling living cells and biologics within a 2D or 3D construct [60]. Sterolithography was the first 3D printing method developed by Charles W. Hull in 1986 to create a solid 3D object [61] and scaffolds that could be used for transplantation and made of biological materials were fabricated using this process [62]. With the many advances if AM technology, 3D functional tissue engineered constructs and scaffolds were then developed to restore organ and tissue function. In what follows we review the different AM techniques, and then we will discuss the conventional methods and the rapid prototyping methods used to manufacture metallic scaffolds.

### 4.1. Categories of Additive Manufacturing

Rapid prototyping can be classified into two groups: substractive or additive. The substractive methods consist of building objects by successively removing material from a work piece. CNC machines are typically used in substractive manufacturing [63]. In additive manufacturing parts are built by successively adding material in layers to form the final shape of the product.

ISO/ASTM 52900:2015 [64] defines the following seven categories of AM technology: (1) Vat Polymerization, (2) Material Jetting, (3) Sheet lamination, (4) Binder Jetting, (5) Directed Energy Deposition, (6) Powder bed fusion, and (7) Material extrusion. The main difference between the various AM methodologies is the way of producing the individual layers which are typically about 0.1 mm Table 2 [65].

#### 4.1.1. Vat Polymerization and Stereolithography

The term Vat Polymerization is a general term that encompasses stereolithography [66]. In this method, a liquid photopolymer is cured into a specific shape. A platform is moved up and down inside a container that is filled with photo curable liquid-acrylate polymer. A photonitiator is included in the liquid. Using an ultraviolet beam, a laser is used to cure and produce a layer. The platform is then lowered and layers are subsequently produced. Stereolithography does not require strong support material.

#### 4.1.2. Extrusion Based Systems

Extrusion based technology is very popular on the market. Heat is used to melt material. The fuel deposition modeling (FDM) is the most common extrusion based system. FDM has been employed in the fabrication of 3D scaffolds. In this process, an extruder head moves over a table that can be moved up and down. The extruder head is heated and extrudes a thermoplastic polymer filament. The table is lowered after the first layer is completed and the next layer is superimposed on and bonds to the previously deposited one. So, molten polymers or ceramics are extruded though a nozzle with a small diameter and merged with the material of the previous layer. In this method, a support material is extruded allowing the layers to be supported by the material under them. The extrusion die diameter determines the thickness of the extruded layers.

Three-dimensional scaffolds using polymers such as polylactic acid (PLA) [67], polyether ether ketone (PEEK) [68], polycaprolactone (PCL), high-density polyethylene (HDPE) and composites such as PLA/amorphous magnesium phosphate [67] and PCL/hydroxyapatite have been made using the FDM technique. Pore sizes up to 700 microns with porosities up to 75% can be achieved using the FDM method. PCL scaffolds made using FDM were found to have a compressive stiffness ranging from 4 to 77 MPa replicating thus the mechanical stiffness for both soft and hard tissues [69].

Metal wires can be used instead of polymer filaments. In this case, a laser is needed to heat and bond the deposited wire while building the part. In this method, surface roughness is a problem as a stepped surface exists in the build part. Fused deposition of ceramics, which is a modification of the FDM, has also been developed for fabrication of scaffolds from B-tricalcium.

#### 4.1.3. Material Jetting

Material Jetting was developed by Israeli 3D printer manufacturer Objet Geometries in 1998. Both PolyJet and Material Jetting are the same technology where Multijet/PolyJet Modeling is the name patented, whereas Material Jetting is the technical name for the process. In this method, print heads deposit the photopolymer on the build tray. Layers are cured instantly using ultraviolet bulbs in conjunction with the jets. Smooth surfaces are obtained using this method as thin as 20 µm [70]. Supports are required in this process, and are printed concurrently with the part. Supports are usually made from a different material that can separate from the part when dissolved in water. In this technique, two separate materials are thus jetted and cured simultaneously. The first material is to build the part. The second material is a gel-like resin and is used for support. The support material is removed with an aqueous solution. In this technique, all the print material is dispensed from a print head. Print head speed and droplet frequency and size affect the quality of the produced part. Thin layers are produced that are cleaned up easily reducing thus the post-processing time. Polymers, ceramics, and metals are used in material jetting.

#### 4.1.4. Powder Bed Fusion

Powder bed fusion (PBF) processes include a thermal source to fuse powder particles, a method to control powder fusion to confined region of each layer and a mechanism for adding powder layers [66]. Selective laser sintering (SLS) was developed at the University of Texas, Austin, USA, as the first commercialized PBF process. In the SLS method, nonmetallic and metallic powder is sintered to form an object. The system can be characterized as having a process chamber whose bottom is outfitted with two cylinders: the powder-feed cylinder moves up to supply the powder to the second cylinder, the part-build cylinder which moves down. A laser beam is focused on the layer of powder deposited in the part-built cylinder to sinter a specific cross section. The remaining unsintered powder supports the sintered potion. In this process, and except of ceramic, the part does not require further curing as the loose particles are brushed off the part. Wax, metals, ceramics, polymers such as ABS, PVC, PBF (nylon), polyester, polyetyrene can be used in this process [70]. Polymer binders that have been mixed with ceramic and metal powders are also sintered. Stainless steel, titanium, and its alloys, and cobalt-chrome have been processed using SLS.

The following fusion mechanisms are used in the PBF processes: solid-state sintering, chemically-induced binding, liquid phase sintering, and full melting. The mechanism of sintering in the solid-state sintering is diffusion between powder particles. The chemically induced sintering is utilized for ceramics materials where two types of powders or powders and atmospheric gases react with each other using a laser causing the powders to bind together. In the liquid phase sintering (LPS) a portion of components within some powder particles become molten while other components remain solid; the molten components bind the solid particles together. In the LPS systems, binding and part material are different. They can be separate, composite, or part material coated with the binding material. In the full melting fusion mechanism, the material is melted to a depth that exceeds the layer thickness using a laser or an electron beam.

#### 4.1.5. Binder Jetting (3D Printing)

3D printing was the common name for binder jetting and was invented at the MIT in the early 1990s. 3D printing most commonly refers to printing a binder onto a powder bed to form a cross section. In this method, a print head deposits an inorganic binder material onto a layer of metallic, ceramic, or polymer powder [70]. The powder bed is lowered and a new layer of the binder is deposited to fuse a new layer of the powder. Metals and blends of polymers and fibers are the most common powder materials. Common metals used in 3DP are stainless steels, aluminum, and titanium. It is possible to produce colored prototypes by using different binders with different colors. Binder jetting is faster than material jetting. However, parts made using binder jetting have poorer accuracies and surface than similar parts made of material jetting.

#### 4.1.6. Sheet Lamination

Several sheet lamination techniques exist. The initial technique is the Laminated Object Manufacturing (LOM) method where adhesively paper or plastic bonded layers to one another are laid down. A laser is used to create the shape by burning it into a sheet and a heat activated glue is used to bond the layers. Recently, Ultrasonic Additive Manufacturing (UAM) was identified as a sheet lamination process. UAM combines ultrasonic metal seam welding and CNC milling.

#### 4.1.7. Directed Energy Deposition (DED)

In this method, parts are created by melting materials are melt as they are being deposited (not materials prelaid in a powder bed such as in powder bed fusion [66]). Laser or electron beam are used in the DED processes. In the Laser Engineered Net Shaping (LENS) technique, metal powder or wire is melted and deposited over a previously molten layer. A laser beam is used for this purpose. In the Electron-beam melting method, metal prototypes are fabricated by melting titanium or cobalt-chrome powder using an electron beam. Electron beams are more efficient than lasers from an energy point of view [70].

### 4.2. Conventional Methods to Fabricate Metallic Porous Scaffolds

Several conventional methods have been reported to fabricate metallic scaffolds [71]. These methods can be classified in producing products with open-cell and closed-cell porosities. The pores are surrounded by a metallic barrier in a porous structure with a closed-cell porosity. Open-cell structures include interconnected pores. The gas injection into the metal melt [72] was associated with a closed-cell porosity and had a random pore distribution with a porosity up to 76%. The decomposition of foaming agent technique [73] was also associated with a closed-cell porosity and produced a Ti-6Al-4V structure with a porosity up to 80% using a powder metallurgy process where the TiH2 was employed as the pore forming and active agent. Several other conventional methods were associated with open-cell porosity. The sintered metal powders and sintered metal fibers [74,75] techniques were able to achieve a porosity ranging from 20% to 80% and produced an open-cell scaffold with a non-homogeneous pore distribution. The spark plasma sintering (SPS) technique [76] produced a scaffold with a porosity ranging from 50% to 60%. The fiber meshes sintering technique [77] produced a scaffold with a homogeneous porosity less than 90%.

All the above conventional methods to manufacture metallic scaffolds have limitations as pore size, pore geometry, pore interconnectivity, and cannot be precisely controlled. Furthermore, the ductility of porous titanium and its alloys are highly reduced as they are affected by atmospheric gases such as oxygen and nitrogen [78].

### 4.3. RP Methods to Fabricate Metallic Porous Scaffolds

The different rapid prototyping techniques that have been identified to manufacture metal and Ti alloy scaffolds include three dimensional printing (3DP), sacrificial wax template, 3D fiber 391 decomposition technique (3DF), electron beam melting (EBM), selective laser melting (SLM), direct metal decomposition (DMD), laser-engineered net shaping (LENT), and selective laser sintering (SLS). These techniques can thus be classified as (1) inkjet based, (2) laser-light based, and (3) extrusion based [79,80]. Each of the above RP methods has special characteristics with its advantages and disadvantages.

**Figure 3:**
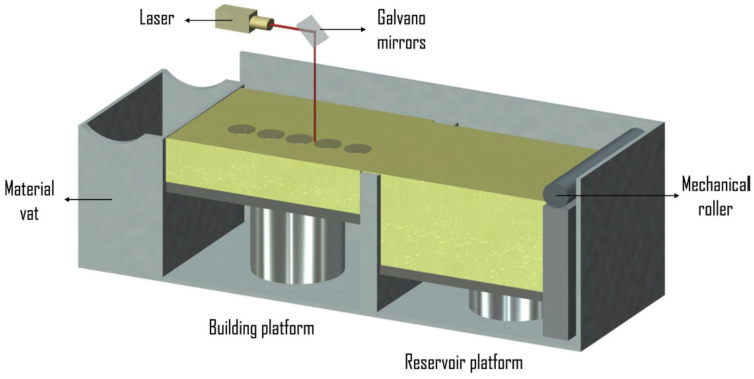
Schematic of the SLS Process [85].

The SLS method consist of a laser, powder bed, a piston to move down and a roller to spread a new powder layer [81,82]. The powder is sintered by the laser beam and the untreated powder serves as a support for the structure being built. Ti-6Al-4V materials with variable porosities mimicking human trabecular bone were produced using SLS [83]. A flexible interconnected porous design and a fine resolution were achieved when the SLS method [84] was used to fabricate Ti alloy scaffolds; however, post processing was required to increase the density. Figure 3 shows a schematic of the SLS process [85].

**Figure 4:**
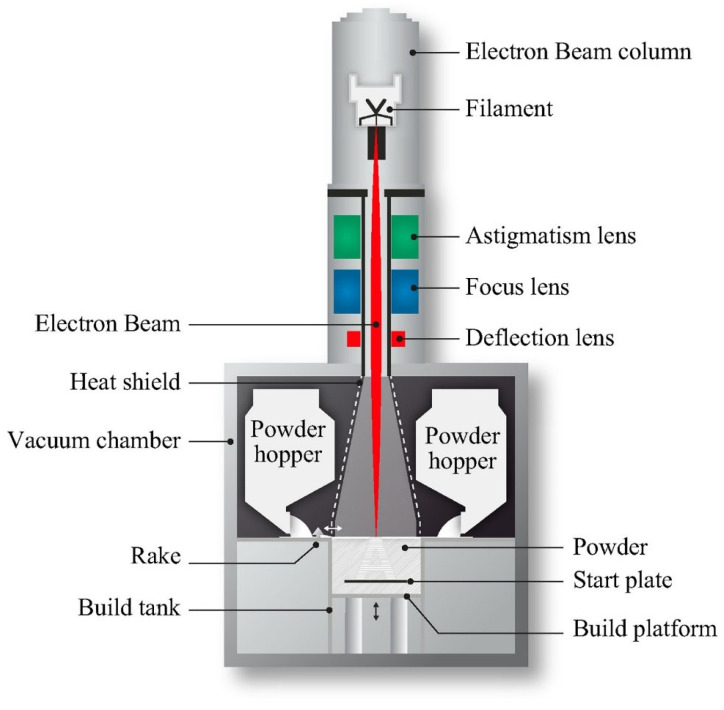
Schematic of the EBM process [87].

The EBM technique is fast but is costly with low surface quality and dimensional accuracy [86]. The EBM method includes two compartments, which are kept in a high vacuum: an electron beam gun compartment and a specimen fabrication compartment. A high energy electron is used to melt the metal powder. The 3DP was used for CoCr alloys, Ti, Ti alloys, and stainless steel scaffolds where independent control of porosity and pore size can be achieved. Figure 4 shows a schematic diagram of the EBM process [87].

The SLM technique allows using a large variety of materials in the form of powder [88]. However, it is costly and it is difficult to remove the unbounded powder from the structure. The LENS technique is also costly [89]. The 3DF technique for Ti and its alloys was reported to provide high surface quality and dimensional accuracy [90] but with low resolution. The DMD technique allows for a fabrication of a near-net-shape scaffold made of Ti and its alloys with good surface finish; a disadvantage of this technique is that it involves multi steps to achieve the required strength and ductility [91].

## 5. Materials for Am Metallic Scaffolds

Tantalum, Magnesium, Titanium, Nickel-Titanium alloys, and hybrid constructs have been tested in-vivo and in-vitro to assess their biocompatibility as bone scaffolds. In what follow we discuss some of the preclinical and clinical trials using these metallic scaffolds.

### 5.1. Tantalum

Porous Tantalum has been used in total hip arthroplasties and to provide structural support of osteonecrosis among other lesions. The efficacy of porous tantalum scaffolds has been tested in preclinical trials. Zhang, et al. [92] reported that the coefficient of friction of Ta porous scaffolds was higher than that of bovine cortical or trabecular bone. Using a canine model, Bobyn, et al. [93,94] reported Ta porous scaffolds had good porous architecture to allow for 63% to 80% of bone ingrowth by 52 weeks. Hacking, et al. [95] implanted porous TA scaffold in the back muscle of dog and reported normal fibrous ingrowth. Rahbek, et al. [96] reported that porous TA allowed for excellent bone ingrowth when implanted into the knee joints of dogs. Adams, et al. [97] implanted cylindrical dowels of porous Ta into a defect created at canine carpal bones and found good bone in growth by 4 weeks. Zou, et al. [98] also observed bone ingrowth when porous TA was implanted in a porcine lumbar inter body fusion model.

Tantalum has also been tested in clinical trials. Long, et al. [99] and Meneghini, et al. [100] implanted porous TA seal cones into patients with total knee arthroplasty and found them to have stable bone ingrowth and good osseo-integration. Nadeau, M. et al. [101] reported overall 44.5% success rate when porous TA plugs were implanted into 15 patients with advanced stage of osteonecrotic hips. Tsao, et al. [102] conducted a similar study where porous TA plugs were implanted into 98 patients with early-stage osteonecrotic hips and found that the average Harris hip score increased from 63 preoperatively to 83 after 4 years. Dursham, et al. [103] implanted tantalum mesh to repair a large cranial defect (greater than 25 cm^2^) into 8 patients where 25% of the cranioplasty got infected and had to be removed. Shuler, et al. [104] also implanted TA plug into 24 patients with early stage hip osteonecrosis and reported that the porous TA scaffold is effective and safe for femoral head salvation.

### 5.2. Magnesium

Only in vitro [105] and preclinical studies using animal models were directed towards studying the effects of using Mg as bone scaffolds. Magnesium has a fast degradation rate, which can be slowed down using surface modification [105]. Reifenrath, et al. [106] implanted magnesium alloy AZ91 open porous scaffolds into the medial condyles of rabbits. Reifenrath, et al. reported that the scaffolds degraded fast and the necessary subchondral bone was not formed. Witte, et al. [107,108] conducted a series of studies by implanting magnesium alloy AZ91 into the patellar cartilage, distal femoral condyle, and the condyles of rabbits, respectively. They reported rapid degradation of the scaffolds with a good biocompatibility.

### 5.3. Titanium

It has been reported that porous titanium and titanium alloys have excellent mechanical properties under load-bearing conditions [71]. The interconnected porous structure of Ti foams allow bone in growth for bone augmentation and marginal bone defects [109]. Also, titanium fiber-mesh is a convenient material for scaffolds and a promising tool for surgery of bone reconstruction. Titanium fiber-mesh scaffolds in vitro help for adhesion and osteoblastic differentiation of progenitor cells [110], while acting as an osteoconductive material in vivo [111].

Results of the preclinical studies confirm that healing of bone is possible using biochemically modified Ti scaffolds, specifically by the use of growth factors and osteoprogenitor cells. Chang, et al. [112] implanted fiber meshes fabricated by sintering and plasma spraying into femoral defects in dogs and reported complete osseointegration due to the abundant bone ingrowth. Matsuzaka, et al. [113] implanted Ti porous scaffolds fabricated by space holder technique in rat femur and reported new bone tissue formation around the scaffold after two weeks implantation. Ponader, et al. [114] implanted porous Ti6A14V scaffold fabricated by selective beam melting (SEBM) into defects in the frontal skull of pigs. They reported that the scaffold allowed bone ingrowth (about 46%) within 60 days. Li, J.P. et al. [115] also implanted porous Ti6A14V scaffold made by 3D fiber deposition into the posterior lumbar of spine goats and reported that the porosity and the pore sizes greatly affect bone formation. Bottino, et al. [116] implanted powder metallurgy processed Ti13Nb13Zr porous samples into rabbit tibia but did not observe bone ingrowth due to the pore structure and distribution. Lopez-Hereida et al. [117] implanted a titanium scaffold made by rapid prototyping into the femoral epiphysis of rabbits and observed about 24% of bone ingrowth after 3 weeks. Takemoto, et al. [118] implanted porous Ti with a bioactive titania layer fabricated by the spacer method into the anterior lumbar spine of dogs and reported inter body fusion in all dogs. They conclude that the bioactive titania improve bone-bonding and fusion of the Ti scaffolds. Pinto-Faria, et al. [109] implanted porous Ti sponge rods made by the space holder method into the humerus bone of a canine model. They reported that the Ti foam has good compatibility and a good bone-growth distribution.

The reconstruction of large anterior column defects has been successful when cylindrical titanium meshes have been used. It has been noted that a good axial load-bearing capacity has been observed following synthetic cages implantation [119]. Eck, et al. [119] implanted titanium mesh cages into 66 adult patients with sagittal deformities. They reported that these Ti mesh cages maintained sagittal correction and provided an average segmental improvement in lordosis of about 11 degrees. Van Jonbergen, et al. [120] implanted titanium SynCage C filled with autogenous bone graft into 71 patients (23 to 76 years) with cervical spinal stenosis and cervical disc disease. They reported that fusion was achieved in all patients but they also noted a disturbing subsidence behavior of the cage design. Kuttenberger, et al. [121] implanted a laser-perforated titanium micro-mesh into 20 patients (22–78 years old) with defects in the craniofacial and/or orbito-ethmoidal region. After 8 years follow-up, they reported that the stable reconstructions of these complex anatomical structures was achieved. Bystedt, et al. [122] implanted porous titanium granules into 16 patients (53 to 83 years old) to augment the sinus floor and reported that they function well. Jaquiery, et al. [123] implanted titanium meshes into 26 patients (13 to 82 years old) with small and mid-size orbital defects. They demonstrated that titanium meshes can support the orbital content, as 91% of the patients had normal vision postoperatively. Scholz, et al. [124] implanted individually fabricated CAD/CAM titanium porous plate into a 16-year-old patient experiencing a severe head injury with an intracranial hematoma. They used successfully CAD/CAM porous titanium plate to reconstruct large bone defects in the skull. A larger group of patients with extended follow-up periods are needed to establish reliable clinical success rates for these scaffolds.

### 5.4. Nickel-Titanium Alloy (Nitinol)

Nitinol (AKA NiTi, a Nickel Titanium alloy) is one of the most well-known biocompatible Shape Memory Alloys. Shape Memory alloys in general exhibit two interesting properties: (1) Super-elastic Effect (SE): the ability to recover large deformations (i.e., up to 10–15% strain) upon unloading, and (2) Shape Memory Effect (SME): the ability to recover large permanent deformations after heating. SME and SE are created due to a reversible solid state Phase transformation between the two phases of Austenite and Martensite. These two interesting properties as well as biocompatibility, made Nitinol a great candidate for a wide range of biomedical applications [125]. Nitinol with SME has been used for developing compact actuator mechanisms in some biomedical devices such as Rectal Retractor [126]. Nitinol with SE properties has been used in several biomedical applications such as cardiovascular stents and artificial heart valves [125], bone implants [127,128], ankle foot orthosis (AFO) [129], and orthodontics arch wires [130].

Recently, additive manufacturing techniques, such as selective laser melting (SLM) has been investigated by different research groups for fabricating Nitinol with both SE and SME [131,132,133]. Additive manufacturing of Nitinol has the potential to introduce revolutionary and pioneering applications, such as stiffness-matched patent specific bone implants [125]. Jahadakbar, et al. showed by introducing engineering porosity and lattice structures based on CT scan data to the bone implants, stiffness modulated porous NiTi implants can be fabricated via additive manufacturing [134,135,136]. Additive manufacturing of Nitinol has also been proposed for fabricating patient-specific cardiovascular implants and joint replacement implants. Thermomechanical properties of the AM fabricated Nitinol parts, such as mechanical fatigue, has also been studied [137,138] to some extends.

Only few preclinical trials using animal models have been carried out about using porous Nitinol as scaffold material. Ayers, et al. [139] and Kujala, et al. [140] implanted NiTi porous scaffold fabricated by self-propagating high temperature synthesis (SHS) with different pore sizes an different porosity into cranial defects in rabbits and femoral defects in rats, respectively. They both reported that the scaffold allows bone ingrowth. Shishkovsky, et al. [84] implanted porous NiTi scaffold made of selective laser sintering (SLS) and SHS into dextral blade bone of rats and reported no bone resorption and biointegration. Zhu, et al. [141] implanted porous NiTi scaffold made by element powder sintering into the femur of rabbits and reported bone ingrowth with good osteocondutivity and osseointegration. Rhalmi, et al. [142] implanted porous NiTi inter body fusion device (IFD) into the spinal canal of rabbits and demonstrated that the NiTi was safe as inflammatory response was minimal. Wu, et al. [143] implanted a hydrothermally treated 3D porous NiTi scaffolds fabricated using the capsule free hot isostatic pressing method (CF-HIP) into the femurs of rabbits and demonstrated that bone tissue could grow into the pores of the scaffolds.

Clinically, NiTi meshes have been limited to few studies related to cardiovascular and thoracic surgery. Wang, et al. [144] implanted superelastic porous Nitinol expandable cages into patients with total hip arthroplasty. They reported that the cages provided support in the necrotic femoral head. Arsenova, et al. [145] implanted porous Nitinol scaffold saturated with bone marrow into patients with endoprosthetic bone defects and reported good integration.

### 5.5. Hybrid Constructs

Better bone remodeling has been observed when the surface of Ti and Ta was modified changing the surface topography. Preclinical and clinical studies have been conducted using hybrid Ti and Ta constructs made of titanium-ceramic, titanium-polymer or cell loaded titanium scaffolds to determine their efficacies.

Zhang, et al. [146] implanted silicon-substituted hydroxyapatite (Si—HA) coated-porous Ti made by fiber sintering into the femora of rabbits and reported high bone in growth as the coating appears to greatly improve the surface bioactivity of the porous Ti. Lopez-Heredia et al. [147] implanted Ti scaffolds coated with calcium phosphate (CaP) and constructed using rapid prototyping into the dorsal subcutaneous pounches of rats and observe mineralized collagen but not mature bone. Sargeant, T.D. et al. [148] implanted Ti6Al4V foam made by hot isostatic pressing (HIPing) into a rat femora defect. The porosity of the foam was filled with a peptide amphiphile (PA) nanofiber matrix. Highly mineralized bone ingrowth was observed around and inside the PA-Ti hybrid implant. This indicates that bone mineralization was achieved by filling the porosity of the scaffold with PA. Kroese-Deutman, H.C., et al. [149] implanted a Ti fiber mesh loaded with platelet-rich plasma (PRP) into a rabbit radial defect and observed bone ingrowth that was in direct contact with the Ti surface after 12 weeks.

The preclinical studies have indicated that bone ingrowth is improved by using hybrid Ti and Ta constructs. Clinical trials have validated this finding. Thalgott, J.S., et al. [150] implanted Ti mesh cages filled by coralline hydroxyapatite (HA) and demineralized bone matrix into 50 patients (28 to 72 years old) and reported that a solid fusion rate of 96% was achieved with this combination. They also implanted cylindrical Ti mesh cages filled with local bone graft into 26 non myleopathics patients (34 to 81 years old) and reported 100% fusion rate [151]. Niu, C.C., et al. [80] implanted a Ti alloy cervical spine cage filled with tricalcium phosphate granules into 54 patients (35 to 66 years old) and reported that successful fusion was obtained in 90% of the cases. Chuang, H.C., et al. [152] implanted Ti mesh cages filled with autologous bone graft and triosite (calcium phosphate ceramics) into 15 patients (19 to 69 years old) and reported some success in reconstructing the anterior column after corpectomy. Hibi, H., et al. [153] implanted one Ti mesh coated with platelet-rich plasma and autologous mesenchymal stem cells in an alveolar left osteoplasty of a 9-year-old female patient. They reported that this tissue engineered scaffold allowed for bone regeneration.

## 6. Characteristics of AM Metals

Metals parts produced using additive manufacturing have properties that are different from those produced using casting or the wrought alloys. These properties include density, residual stresses, mechanical behavior, non-equilibrium microstructure, and crystallographic texture [154].

### 6.1. Density

AM may be the only technique that allows the weight of a part to be reduced by reducing its density [155]. Density is affected by the development of pores or by unmelted powder during the fabrication process. The size of the particle of the powder affects the printed part. While large particles help powder spreading, fine particles improve packing density of the powder, resolution, and surface quality. It is known that the powder density is approximately 50% lower than the density of the corresponding bulk material [155]. It has been reported that the porosity increases as the scanning speed increases and/or the laser power decreases, which affects the as-built density. It has also been reported that increasing the laser beam diameter or the powder layer thickness can cause a density loss as high as 10% [156]. Controlling the laser power and the scanning speed can result in high as-built density [156]. Some of the parts obtained using SLM result in 99% density, which can be achieved by optimizing the scanning speed and the laser power.

AM enables fabrication of the parts with engineered porosity. Engineering porosity in contrast to the process pores, are designed by the engineers and are imposed to the CAD files. These porosities are in the form of lattice structures. Engineering porosities reduces the stiffness of the fabricated parts and allow fabrication of stiffness modulated implants. Stiffness-modulated implants reduce the stress shielding, which leads to bone resorption and failure of the bone implants. In addition, engineering porosities enable bone ingrowth (AKA Osseointegration) and improve the connection of the implant with the adjacent tissues.

### 6.2. Residual Stresses

Some common defects in the production of metal AM components include: porosity, poor surface quality, cracking and residual stresses [155]. The thermal residual stresses produce cracking. In Selective laser melting methods, these residual stresses are generated due to the large temperature gradients and rapid solidification, which result in the shrinkage of the melt pool. High thermal residual stresses can also be caused by low scan speeds and a high energy input. In general, metal parts produced using laser beams can exhibit higher residual stresses in comparison to the AM parts fabricated via EBM. The reason is due to the fact that EBM process occurs at high temperatures that reduces the temperature gradient. Warping occurs if the residual stress is greater than the yield stress of the material, and cracking may occur if the residual stress is greater than the ultimate tensile stress of the material. Residual stresses can be predicted using finite element solutions along with computational fluid dynamics [157,158,159]. Also, more details about porosity and residual stresses can be obtained using X-ray computed tomography (XCT). Residual stresses have a large impact on fatigue life. Residual thermal stresses can be reduced by heat treatment. Isothermal heating can also increase the density. Residual stresses are found in as built parts using the SLM method because of the high cooling rate. The ductility of scaffolds is greatly reduced by the residual stresses. Heat treatment is thus used in SLM built parts to relieve the residual stresses.

### 6.3. Mechanical Behavior

It is expected that the strength will increase and the ductility will decrease in AM metal parts built parts compared to those produced using conventional wrought alloys. This is because of the refined microstructure of metals produced using AM. Wrought aluminum is when the metal is worked in the solid form with the help of specific tools [160]. Post treatment improves the mechanical properties of AM built parts. It was found that isothermal heating increased the density resulting in a three-fold increase in the compressive strength of scaffolds from 6 to 18 MPa [82]. It is also reported that post-heat treatment does not only increase the compressive strength but also causes a decrease in the surface roughness and shrinking of pores. On the other hand, chemical etching caused a deterioration of the mechanical properties of porous Ti scaffolds [82].

Tensile and fatigue properties are main mechanical properties evaluated for AM fabricated Ti64 samples. In general, AM fabricated Ti64 samples show high tensile strengths, but poor ductility. The elongation is generally below 10%, the 0.2% YS could reach up to over 1 GPa, and the UTS up to 1.2 GPa. The high strength in the AM fabricated parts (SLM) is attributed to the fine martensitic microstructure. The AM fabricated Ti64 samples via SLM show anisotropy in tensile properties, with horizontally built samples generally showing higher tensile strengths than the vertically built, but lower elongation than the vertically built samples. The fatigue life of the AM fabricated Ti64 samples in as-built condition has been shown to be inferior to the conventionally fabricated parts. The reason is mainly due to the micro pores and micro defects that exist in the SLM fabricated parts. However, post procedures such as HIP has can significantly increase the fatigue life of the AM fabricated metallic parts.

### 6.4. Non-Equilibrium Microstructures

Vastola, et al. [161] used ABAQUS to develop a model that shows the microstructure evolution for the Ti6Al4V alloy during SLM and EBM by implementing the non-equilibrium phase formation and dissolution in an AM modeling framework. The solidification processing of alloys is highly affected by the cooling rate. A non-equilibrium solidification process is due to a high cooling rate. High cooling rates are encountered when using SLM techniques to build parts using AM. This results in non-equilibrium microstructures [162].

#### Crystallographic Texture

Many components are fabricated from materials that have preferred crystallographic orientation, or texture. Strong crystallographic and morphological textures are expected in SLM metal built parts because of the directional solidification and the rapid cooling rates [162]. The scan direction during deposition affects the texture. Interactions between the non-equilibrium microstructure and crystallographic texture affect the mechanical properties of metal built parts. Crystallographic features include grain boundaries. Fatigue life is affected by pores depending on their relative distance from each other and their location with respect to the crystallographic features. It has been recently reported the crystallographic texture of metals can be controlled during SLM as the layer by layer process of SLM can lead to strong crystallographic textures [162].

## 7. Mechanical Characterization of AM Metals (Application to Scaffolds and Medical Implants)

Printed architecture consisting of polyhedral unit cells are popular with selective laser sintering (SLS) or EBM or traditional powder bed binder-jetting 3D printing. The edges of the 3D polyhedral unit cells are represented by struts and are typically cubic, diamond, octahedron, and rhombic dodecahedron in shape [163].

CAD methods are used for topological design and optimization. Optical microscopy (OM) and scanning electron microscopy (SEM) are usually used to characterize the mesh struts of Ti-6Al-4V alloys printed using EBM. CT scans are typically used to analyze pore defects. Compression specimens are usually made in accordance with ASTM E9 for compression testing of metallic materials [53]. High cycle compression fatigue tests are usually conducted according to ASTME466-07 [53]. The compression fatigue tests are usually monitored and recorded in a digital video in order to obtain detailed information of macroscopic damage propagation of specimen.

The literature on the structure-function relationships of Ti-6AI-4V scaffolds is summarized by Kelly, et al. [163]. For scaffolds printed with EBM using polyhedral unit cells:

1. Compressive properties increase in strength with increasing density.
2. Compressive properties increase in strength with increasing energy input.
3. The compressive fatigue strength of the struts declines with more pores in the struts [53].

For scaffolds printed with SLM using polyhedral unit cells:

1. Compressive properties increase in strength with a decrease in strut length to diameter ratio.
2. Cubic unit cells had superior fatigue strength.

### 7.1. In-Vitro Studies

**Figure 5:**
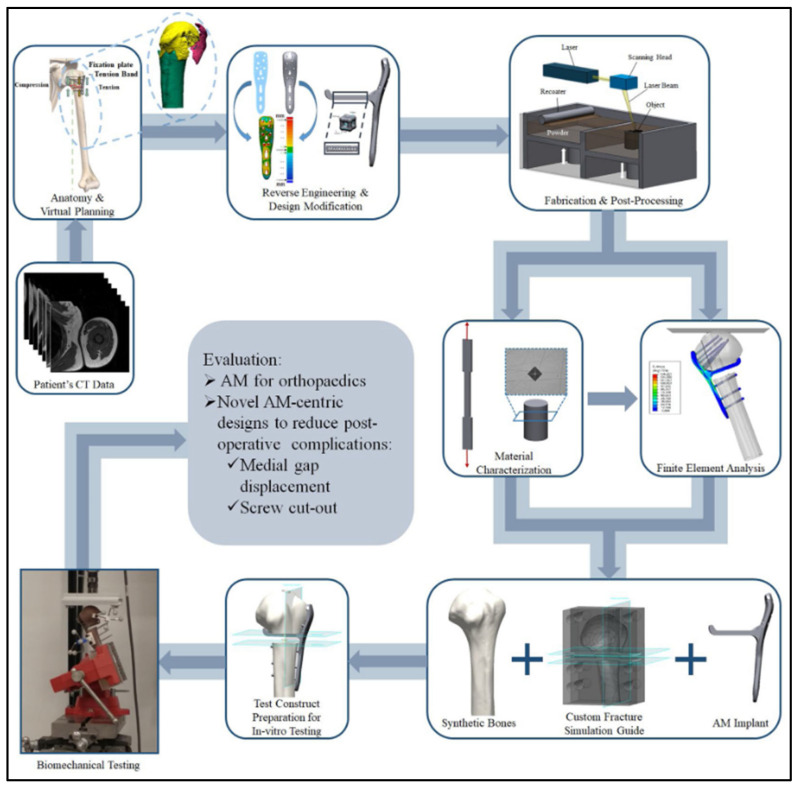
Workflow to evaluate patient specific AM fabrication of fracture fixation implants [164].

In-vitro studies to determine the biomechanical behavior of bone scaffolds can be conducted using normal or composite bones with segmental defects. Biomechanical testing varies with the structure to be tested. In a recent study, Tiltona, et al. (2020) developed a systematic workflow to evaluate patient specific AM fabrication of fracture fixation implants [164]. The workflow shown in Figure 5 includes (i) patient’s CT data, (ii) anatomy and visual planning, (iii) reverse engineering and design modification, (iv) fabrication and post processing, (v) material characterization and finite element analysis using synthetic or cadaveric bones, (vi) test construct preparation for in-vitro testing, (vii) biomechanical testing, and finally (viii) evaluation of the AM implant to come up with a new design.

The schematic in Figure 5 depicts a long femur. Long femurs are mounted into the testing machine and loading conditions are applied to the femoral head to typically simulate the loading during normal walking gait cycle [165]. This review indicates that there are limited biomechanical studies identified in the literature to evaluate Ti-6Al-4V scaffolds. One of these studies was conducted on the stem component of a hip implant printed via L-PBF and using tetrahedron unit cells; the stress shielding was reduced by 75% when compared to fully solid implants.

### 7.2. In-Vivo Studies

During in-vivo studies, animals are anesthetized and incisions are made to insert the scaffolds [166]. Fluorescent dyes calcein are typically injected intramuscularly to assess bone in growth. Animals are then sacrificed typically 2, 4, or 8 weeks after surgery. Bones with porous implants are then either harvested for push-out tests or fixed for further histological analyses. Implants are pushed out of the bone tissues at a specified strain rate to record the maximum push-out stress during the push-out tests.

In-vivo evaluation includes osteogenic differentiation, which is responsible for developing new bones. For the qualitative analysis, the bone in growth area at different time periods is usually observed with a microscope, and digital images are typically analyzed with image analysis software. The dynamic bone ingrowth process and osteogenic pattern is typically quantified by imaging the fluorescently labeled sections with a laser confocal microscope. A porous scaffold avoids stress shielding by having a lower stiffness than normal implants. The metabolism of the cells benefits from interconnected pores. More important, bone regeneration benefits from the porous structure because of the larger space for bone ingrowth they possess. An important conclusion is that different topology designs provide a balance between mechanical function and biological performance.

## 8. Post AM Treatment Applied to Scaffolds

The process of manufacturing and fabricating porous scaffolds entails the design of the scaffold, additive manufacturing 3D printing, and post-treatments. Heat and surface treatment are the two common post-treatments related to AM-derived scaffolds. The last step in manufacturing printed parts is thus post-processing. This step is important because it improves the quality of the printed parts and it enhances their surface characteristics and improves their aesthetics and their geometric accuracy [167]. This step includes smoothing the printed parts that primarily surface irregularities such as the staircase effect, which is created by the nature of building in layers. There can also be layer distortion, balling melts, surface pores, liquid splash, unmelted powders, and surface cracks depending on the exact method of manufacturing implemented [168].

Internal and external surface finishing are required. Mechanical, chemical, and thermal surface finishing techniques are identified as demonstrated in the chart depicted in Figure 5 [168]. Mechanical finishing is the most diverse with a wide number of finishing options. One of the most important types is abrasive flow machining. This came about because there were no options to do surface finishes of the internal channels. Viscoelastic fluids with abrasives are commonly used to polish the micro channels of numerous components under high amounts of pressure. This can reach 220 bar within these channels [168]. Another example of mechanical finishing is magnetic abrasive finishing or MAF. This allows for a very precise surface roughness of nanometers. Iron particles are used and mixed with a wide variety of abrasive particles. These can include pneumatic vibrators along with magnets. This can result in external and internal finishes. Another mechanical finishing process is abrasive fluidized bed machining. This also allows for finishes of external and internal surfaces. The part is placed within the bed while the abrasives are driven by air from the bottom until there are air bubbles. This allows the abrasives to hit the surface of the part [168].

A different category of surface finishes is chemical based. There are generally fewer tooling requirements as the part is simply immersed within an electrolyte solution. DC power is then applied to electrodes to polish the part [168]. Another major category is thermal surface finishing. This usually involves some form of laser polishing. It is unique in that it does not remove any material and melts the surface to rearrange the surface through solidification [168].

**Figure 6:**
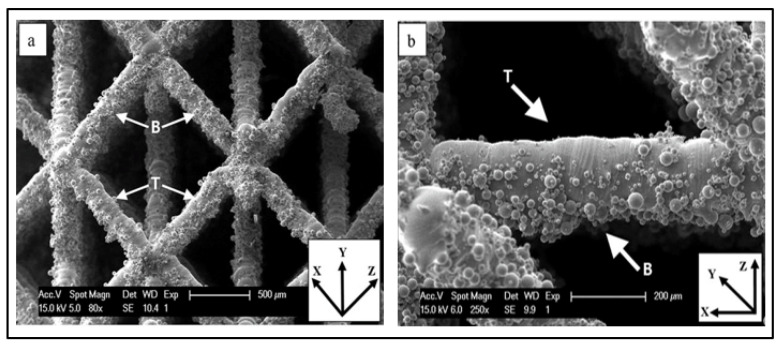
Microscopic images of cell struts: (**a**) unit cell; (**b**) strut with non-melted powder grains [169].

Surface modification of open structures requires surface treatment of the inside of the structure as well as its edges. Grinding and sandblasting may thus not provide a homogeneous surface modification throughout the entire structure. Chemical etching and/or electrochemical polishing are two common processes that provide solution to this problem. Both of these treatments utilize acid-based solutions that are able to penetrate a porous titanium scaffold through their various connected pores [169] and create a smooth surface finish for the printed structure. These two treatments were used by G. Pyka, et al. [169] to compare the surface roughness and the mechanical properties of the 3D printed Ti6Al4V open porous scaffolds, pre and post treatments. The Ti6Al4V porous scaffolds were made using the selective laser melting printing process where a large amount of powder residue is left over. Inhomogeneous roughness were identified in the struts [169]. Figure 6 shows a microscopic view of the cell struts [169] pretreatment where one can see the bumps and grooves along each strut. This figure demonstrates the rough surface finish, indicating that the post treatment is necessary. The T and B in the image represent the top and bottom of the cell strut [169]. There was a large difference in the surface roughness between the top and bottom of the struts [169] pre-treatment as the roughness average, ‘Pa’ varied between 7 µm and 12 µm for the top and bottom struts. Surface roughness improved drastically after post processing. However, the average strut thickness varied by about 22% causing an increase in the pore size, which caused a significant change in the unit cell dimensions as well as a significant reduction in the mechanical properties. Porous structures were thus produced.

Post treatment is also necessary to allow titanium to adhere to the bone tissue, since this alloy has limited capability to bind directly with the bone tissue. A recent study in 2021 [170] proposed to enhance the osteogenesis of 3D printed Ti-6Al-4V implants by constructing a hierarchical micro/nano-topography on the surface. Ti-6Al-4V implants were made using Electron beam melting and modified using acid etching and anodic oxidation to manufacture the nano hierarchical structure on the surface [170]. The method consists of combining ultrasonic acid etching with anodic oxidation for surface modification. Residual powders on the surface were removed by the acid etching, which allowed also creating micro pits and groves on the surface. Anodic oxidation was then employed to impose nanotube arrays on the micro-structured substrate. In vitro and in vivo experiments were conducted to assess the efficiency of this surface modification procedure. Both studies showed the proposed surface modification method allowed for the proliferation and osteogenic differentiation. The in vivo study showed a 1.5 times increase in the ratio of bone volume to total volume (BV/TV) post-treatment as well as a remarkable increase in the bone-to-implant contact area.

Chemical polishing is used on SLM products that are created using titanium-aluminum-niobium alloy [171]. It is an effective post processing method due to its ability to remove the excess material and get the loose particles out of the porous structures [171]. The process of chemical polishing includes three overall steps: surface preparation, chemical cleaning, and final cleaning [171]. Surface preparation of the object is done once it has been pulled from the powder bed by removing any of the large particles. This can be done by ultrasonic cleaning or magnetic stirring. The later method of surface preparation is preferred over the former method. This is because ultrasonic cleaning causes over polishing and loss of material. The part is then dried. Chemical cleaning is where the product is dropped in a chemical bath that completely dissolves all of the remaining material that has been left on the product. Lyczkowska, et al. [171] used two Hydrofluoric acid/nitric acid mixtures with different concentrations for this purpose. During final cleaning, the residual chemicals that are left on the part are rinsed off and removed. Final cleaning can also be done by ultrasonic cleaning and magnetic stirring. Lyczkowska, et al. [171] found that a significant reduction of the bath concentration permits better reduction of the surface roughness.

The poor quality of the contact surfaces between the parts and the anchoring supports is another problem that comes up when additive manufacturing titanium alloy by SLM. Cosma, et al. [172] used SLM to print two customized medical implants: one for the maxillofacial area and the second one being a tibia component that has lattice structure. After manually removing the support structures, they tested three post-processing methods: alumina sandblasting, carborundum polishing, and ultrasonication in isopropyl alcohol [172]. The alumina sandblasting used a particle size of 120 μm and had a pressure of 4 bars. The carborundum polishing used carborundum abrasive disks that were used on a micro-motor at 15,000 rpm. The ultrasonication in isopropyl alcohol that used an ultrasonic bath where the implants were set into for 30 min at 35 °C. Scanning Electron Microscopy (SEM) and Energy-Dispersive X-ray Spectroscopy (EDX were used to determine the effects of these post processing techniques on the surfaces of the manufactured implants). Roughness was reduced from 6.6–8 μm to 1.2 μm. Giving the implant a smooth and fine surface finish allows the body to be more accepting of the implant and for more new bone formation.

Other post processing methods were implemented on Ti6Al4V scaffolds. Ultrasonic vibration post-treatment was looked at specifically to see if it could be a viable alternative to other methods like traditional powder recovery systems [PRS] [173]. The scaffolds that used USV had more partially fused powders which is less desirable as it can lead to worse mechanical properties like compressive strength. The main benefit USV has is that it can be done much quicker than PRS. It ends up being inferior overall with significantly worse mechanical properties for scaffolds [173].

In a recent study, Ginestra, P. et al. (2020) [174] presented an interesting study to effects of surface treatments on the reduction of the bacterial adhesion on the surface of implants allowing a better osseointegration. They printed the Ti6Al4V samples using the Selective Laser Melting (SLM) method and analyzed the effects of post-processing using sandblasting and vibratory finishing treatments on the surface and antimicrobial properties of the 3D printed specimens. Two strains of bacteria, *S. epidermidis* and *P. aeruginosa* were used in this study. It was found that both post-processing techniques lowered the bacterial colonization of the surfaces, in particular for the *S. epidermidis*.

Printed Ti6Al4V parts using SLM or EBM have relatively high yield stress of about 1000 MPa and ultimate strength of 1150 MPa and a relatively low ductility of less than 10%. Internal thermal stresses are developed in the structure of the printed part because of the high cooling rates. Increased porosity and high surface roughness result from the printing process. Post treatment allows the reduction of these thermal stresses. It has been reported that heat treatment of SLM produced Ti6Al4V parts caused a significant improvement in their fatigue strength and ductility; this is because the reduction in the thermal stresses [55].

It is also important to note that surface modification is required to improve the biological bond between Ti alloy implants and surrounding bones. “Balling” effect is observed in AM produced porous Ti alloys where powder become small liquid spheres due to the heating by the laser or the electron beam. The “Balling” effect creates a rough surface with loosely connected powder particles that need to be removed by modifying the surface [55].

The sol-gel, electrolytic deposition (ED) and plasma spray (P) techniques have been identified as surface based coating methods that are employed to modify the surface of porous metallic structures [55]. The sol-gel process is a popular low cost and simple but effective coating-based method where oxide coatings are created on the surface of implants. Thin (<10 μm) inorganic coatings are deposited on the surface of implants. Bioceramic coating (CaP) on porous Ti6Al7Nb implants was uniformly deposited on their internal and external surfaces using the sol-gel method. Improved biocompatibility was observed. CaP coatings with several thicknesses have also been produced using the electrolytic deposition (ED) and plasma spray methods. In plasma spray coating a plasma flame is used to spray hydroxyapatite (HA) on the surface of the implant that then solidifies as a coating. HA is a natural occurring compound found in the bone that promotes regrowth and osseointegration, All of these coating methods allowed to improve the bioactivity of the CaP coated Ti6Al4V scaffolds as osteoinductive “bio-units” were produced which is necessary for the repair of bone defects [55].

Bioactivity of the implants can also be improved using corrosion-based surface treatment methods. Corrosive solutions are used to produce a thin oxide layer (from tens of nanometers to hundreds of microns) on the surface of the metal [55]. These methods include acid etching and alkali and anodization treatments. A biologically active bone-like apatite (this is a mineral) layer is formed on the Ti surface using the alkali treatment method. Protective layers are produced on the metal surface when the electrochemical anodization treatments are used. However, the corrosion-based methods have a negative effect on the mechanical strength of the porous Ti alloy scaffolds causing a significant decrease in the ductility of their struts.

## 9. Computational Biomechanics Aspects of Am Scaffolds

### 9.1. CAD for AM Printing

**Figure 7:**
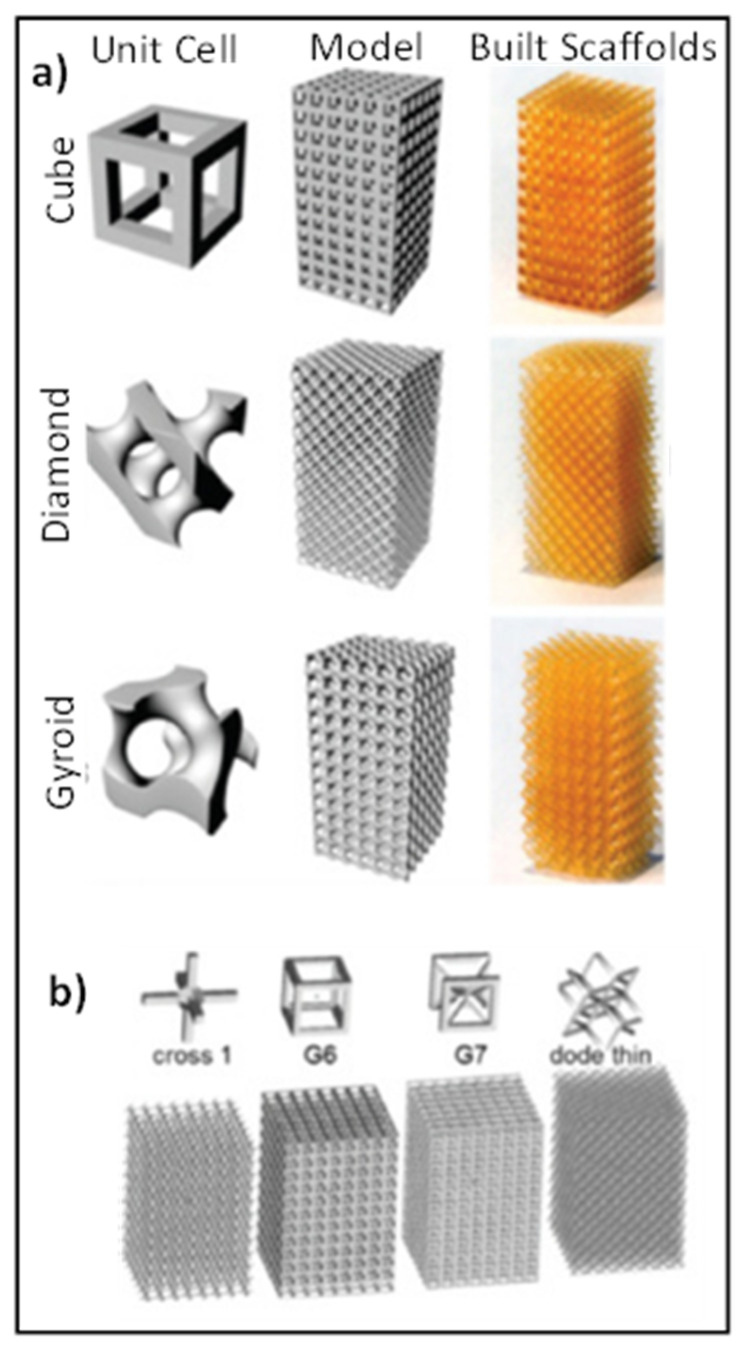
(**a**) CAD based unit cells, their assembled models, and as-built scaffolds; (**b**) Materialize software elements and scaffold models [82].

A scaffold is characterized by its external shape and its porous internal structure. Thanks to the recent advances in computer science and higher computational powers, these days there are many design software packages available for designing scaffolds and lattice structures. In general, these software packages provide the conventional CAD design capabilities, or STL design tools, or a combination of both. In addition to commercial CAD software packages (e.g., Pro/Engineer, CATIA, Solidworks, etc.), STL software packages (e.g., 3-Matic by Materialise, Geomagics, etc.) have been used to design 3D scaffold models and to develop more complex lattice structures and scaffold designs. These tools construct models based on constructive solid geometry (CSG) or boundary representation principle (B-Rep). The CSG method requires much less storage space than the B-Rep method. These CAD tools form various porous unit cells and assemble them to make the scaffold. The cube, diamond and gyroid unit cells were used to build three scaffold models as shown in Figure 7a [82]. Finite element analysis was used to simulate the compressive behavior of each of these scaffolds [82]. Some dedicated CAD software have been developed to simplify this process. The Belgium Materialize Company has developed Magics which is a 3D printing software that allows the integration of various built-in unit cells. Figure 7b shows Materialize software elements namely cross1, G6, G7 and dode thin. This figure also shows the designed scaffold models [82].

### 9.2. Mechanical Properties Simulation

Lawrence Livermore National Lab [175] is working on developing predictive tools to derive the relationships between structure, processing, properties, and performance of powder-bed additive manufacturing (AM) of metals. They used a tetrahedron to represent the key components of their activities. The center of the tetrahedron was “modeling and simulation”. The four corners of the tetrahedron represented each of the activities surrounding modeling: structure, processing, properties, and performance. It has been recognized that developing complex designs using AM is very expensive: money-wise and time wise. Lawrence Livermore is thus currently investing in a strategic initiative to advance the field of AM by developing predictive process-structure-property relationships and by developing a systematic understanding of the basic physics of AM processes. High-performance computational simulations are required to develop the process optimization and simulation modeling. This initiative is based on the work performed by the Institute for Computational and Mathematical Engineering at Stanford University. The purpose of this initiative include developing physics-based models to relate processing and post-processing to microstructure and properties and providing validating experiments. By doing this, they hope to develop new processes to improve the quality and speed of AM.

The modeling efforts to establish process-property-performance predictive connection incorporate length scales of hundreds of micrometers and a time scale of seconds. This represents the challenge that is faced by researchers face when developing process computational models; the problem involves multi-physics and is multi-scale in nature. Integrated models that take into account different scales are conducted at Lawrence Livermore National lab jointly with Los Alamos National Laboratory and include numerical simulation at the microstructural level [176] to determine the causes of failure. Simulations are being conducted to utilize microstructure to explain how a material deforms when stressed. The anisotropic characteristic of titanium alloys can cause damage initiation at the macroscopic level. The work at Lawrence Livermore National Lab to explain and illustrate the low macroscopic damage initiation mechanisms is based on the work by others on the modeling of Ti6Al4V microstructures. This activity represents the first purpose of the computational models, which is to model the AM process itself and to predict the resulting residual stresses. For instance, in an effort to model the AM process itself, Rausch, et al. [177] developed a simulation tool to determine the influence of powder size distribution on surface roughness and porosity. This work is directed at manufacturing parts with a reproducible high surface quality using powder bed fusion additive manufacturing.

The second purpose of developing computational models is to design for AM [178]. Metal is used in most orthopaedic implants and scaffolds that are custom-made. This requires developing specific anatomical models of the parts to be made. Medical images obtained from specific patients are used to build the 3-D models to be used in the design of the implants and scaffolds. Anatomical models are thus required for patient-specific implants.

It is being recognized that a big advantage of AM is its capability of producing open-cell porous implants with a micro-scale specific topological design. Another aspect of the design is thus to use AM to develop new materials, known as metamaterials, to achieve certain unique properties [179,180]. The research related to metamaterials is mainly directed at determining topology-properties relationships. Modeling allows us to determine the effects of a specific topological design Ahmadi, et al. [179] reported that the material type and manufacturing imperfections are more important than the topological design in determining the normalized S-N curves of AM meta-biomaterials. Hedayati, et al. [180] conducted a study to quantify the effects of topological design and material type on the mechanical properties of AM porous biomaterials. They concluded that the material type systematically affects the mechanical properties of AM porous biomaterials.

Review of the literature indicates that the above referenced topology-property relationships are established mostly using simplistic models. These include finite element models [181,182], optimization algorithms [183,184,185,186], and analytical models [187,188,189].

Cho, et al. [181] used the finite element method to perform a structural analysis of periodic cellular materials. The three-dimensional structure of titanium foam formed using selective laser melting (SLM) was obtained using an X-ray microtomography. A dual-level FEM was used to determine the deformation behavior of titanium foam under uniaxial compression. In the classical FEM approach, the material behavior is determined using certain constitutive relationships that employ macroscopic material properties such as Young’s modulus and Poisson’s ratio. Nevertheless, these relationships are extremely affected by the microstructure which changes continuously during loading. A dual-scale FE method was introduced to model the microstructure changes. This methods consists of two steps: an upper-level FE analysis and a lower-level FE simulation. ABAQUS was used to solve the model using user subroutines. Tetrahedral elements were used to model the specific volume based on the microstructure obtained from the X-ray microtomography.

Kadkhodapour, et al. [182] used the finite element method to study the deformation and failure mechanisms of porous titanium (Ti6Al4V) biomaterials manufactured by selective laser melting. Cubic and diamond lattice structures were modelled with different porosities. Failure of the additively manufactured scaffolds was studied under compression using the Johnson-Cook damage model. Lin, et al. [183] used the selective laser melting technique to fabricate lumbar fusion cages made of Ti-6Al-4V. An image-based finite element technique was used to model the microstructure.

Hedayati, et al. [190] developed a sophisticated multi-scale finite element model to study crack propagation in AM porous bio-materials. At the microscale, beam elements were used to model the area around the crack tip. At the macroscale, volumetric elements were used to model the area far from the crack tip. Cubic diamond unit cells were used to model the lattice structure. Specimens were fabricated from Ti-6Al-4V using selective laser melting and tested under fatigue. The multi-scale computational model was successfully used to predict crack propagation in the AM porous biomaterials.

Gradient based schemes for topology optimization (TO) have been used recently to optimize the internal architecture of porous materials. Isogeometric analysis (IGA) was recognized lately to be more efficient that the finite element method. For example, checkerboards can be solved in FEA using higher order elements, which requires high computational power. On the other hand, these problems can be solved using IGA with much lower computational cost. Checkerboard pattern appears when the domain has subdomains consisting of alternating solid and void elements. Wang, et al. [184] presented a framework that uses a multiscale isogeometric topology optimization to optimize the relative density of homogeneous and graded lattices and to calculate mechanical properties. For lattice materials with homogeneous density, the effective stiffness matrix of the element material was expressed as a function of the stiffness matrix of the solid material and the element density. For graded lattice materials, the effective mechanical properties were expressed as a function of the unit cell relative density.

Xiao, et al. [185,186] combined additive manufacturing and topology optimization to design three lightweight lattice structures subject to compressive loading. Face Centre Cube (FCC), Vertex Cube (VC), and Edge Centre Cube (ECC) lattice structure units were obtained by topology optimization of the cube using the ABAQUS software, which integrates the topology optimization module TOSCA. The lattice structures were fabricated using SLM technology. A Gibson-Ashby model was developed to predict the performance of the three structures including different levels of porosity. Xiao, et al. [186] reported that FCC and VC lattice structures have better mechanical behavior compared with that of the ECC lattice structure.

Ahmadi, et al. [191] did an experimental study to determine the relationship between morphological and mechanical properties of different porous titanium alloy biomaterials. They considered six different types of space-filling unit cells. They reported that the response of the porous structures are highly dependent on the properties of the unit cells including relative density. The diamond unit cell had lower compressive properties. The truncated cube, truncated cuboctahedron, rhombicuboctahedron, and cube had a high stiffness while the diamond and rhombic dodecahedron had low stiffness, with the truncated cube having the highest stiffness. Later their group conducted an analytical and a finite element study [187] to estimate the mechanical properties of open-cell porous biomaterials made of truncated cube unit cells. Their results correlated well with the above referenced experimental study. The relative density was defined as the ratio of the density of a porous structure to the density of the solid material that it is made of. The truncated cube lattice structures were considered to be made of struts with circle, square, and equilateral triangle cross sections. The relative density of this structure was defined as the ratio of the volume occupied by the material to the total volume of the unit cell. The elastic modulus and Poisson’s ratio were determined as functions of the area moment of inertia I and cross-section area A for each cross-section type. Yield stress was obtained by assuming first that the structure yields when the stress in the vertical struts equals the yield stress of the matrix. The yield stress of the structure was also obtained by assuming that yielding first occurs in the inclined struts. The elastic buckling limit was also obtained because failure can occur due to buckling as porous biomaterials are usually loaded in compression. The commercial finite element package ANSYS was also used to create finite element models of the porous structures employing beam elements made of Ti6Al4V-ELI alloy. First, a small model was developed consisting of eighteen struts similar to the configuration used for the analytical study. Then, a FE model of the lattice 3D structure was developed using the truncated cube unit cell. Results were obtained to compare the elastic modulus of the truncated cube, diamond, rhombic dodecahedron, and cube. It was found that the truncated cube structure allows a wider range of stiffness for different relative densities. It thus appears to be very relevant in orthopaedic applications where the local stiffness of the bone at different locations are different.

In 2016, Hedayati, et al. [188,189] did similar analytical and numerical studies to analyze porous structures made of rhombicuboactehedron and truncated cuboctahedron unit cells, respectively. They reported that by adjusting the relative density of the porous structures made of truncated cuboctahedron unit cells [189], their mechanical properties were in the range of those of trabecular and cortical bone [192]. They also reported that the elastic modulus of porous structures made of rhombicuboactehedron unit cells [188], and when Ti-6Al-4V is used as the matrix material, is within the values of the elastic moduli of natural bone [193] making them also suitable candidates for bone replacements.

Most of the studies directed at determining the fatigue response of open-cell metal foams [194,195,196,197,198,199] and additively manufactured lattice structures [200,201,202] have been experimental in nature which is costly and time consuming. In 2016, Hedayati, et al. [203] created finite element models to predict the S-N curve of additively manufactured porous biomaterials. Models were obtained for porous titanium alloy (Ti6Al4V-ELI) structures based on rhombic dodecahedron repeating unit cell to analyze their static and fatigue behavior under compression. Their computational results were validated using previously reported experimental data [204]. Similar to their previous studies, they also reported that the elastic moduli of the porous structures were in the range of those of human bones [205,206]. Their fatigue results also show the number of loading cycles had initially a relatively slow rate of change on the elastic modulus and number of failed struts, while this changed very rapidly later. They further reported that the computationally predicted 5-N curve was consistent with their previously described experimental data for stress levels not exceeding 60% of the yield stress of the porous structures. For higher stress levels, their numerical simulation significantly underestimated the fatigue life of the porous structures. The effects of the irregularities caused by the additive manufacturing process on the fatigue behavior of the porous structures were also studied. In this study, Hedayati, et al. [203] also determined the effects of the manufacturing processes and reported that irregularities and initial damages significantly decrease the fatigue life of these porous structures.

As mentioned earlier, Hedayati, et al. [190] proposed a multi-scale computational approach to predict crack propagation in additive manufactured porous biomaterials. The multi-scale model had relatively small computational time, relatively better stress distribution prediction in the crack tip region, and relatively less solution instabilities. However, the preprocessing procedure for the multi-scale model is relatively complex. Hedayati, et al. [190] reported that it is important to consider the plasticity of the parent material. This is because the struts located around the crack tip are usually in the plastic regime.

## 10. Advantages and Limitations of AM Scaffolds

AM has a great potential in tissue engineering applications. AM scaffolds can have complex shapes and can be cellular having lightweight. They are built directly from CT scan data using computer models insuring that the design specifications are realized. There is minimum waste in production since only the material needed for the part is used as material is added layer by layer.

Bone tissue engineering is a complex that involves matrix formation along with remodeling of the bone. Biocompatible porous degradable materials that provide structural support during bone repair are used to make bone scaffolds [207,208]. The scaffold must be osteoconductive allowing bone cells to form the extracellular matrix on its pores. The mechanical properties of the bone scaffold should match those of the natural bones of low stiffness and high strength. Porous scaffolds can be made from polymers, ceramics, and metals. Metals have high compressive strengths. Researchers from the Wyss Institute at Harvard and German colleagues from the Julius Wolff Institute in Berlin, the Berlin-Brandenburg Center for Regenerative Therapies, and Charité’s Center for Musculoskeletal Surgery have recently shown that titanium-mesh scaffolds allow for bone ingrowth and induce bone regeneration. They filled the honeycomb-like scaffold structure with the patient’s own bone tissue and demonstrated that soft implants produced faster bone growth than stiffer implants. The porous metallic scaffolds made of titanium (Ti) and tantalum (Ta) and Ti-6Al-4V alloy powders have been studied as bone replacement materials since they have affordable prices and high compressive strengths. Selective laser melting (SLM), electron beam melting (EBM) and Laser Engineered Net Shaping (LENS) are the most common AM fabrication techniques used to fabricate titanium scaffolds. However, there is a concern about health issues from using titanium alloys because of the release of metal ions into the body. New titanium alloys as well as using reinforcements were thus identified. Ti-6Al-4V-xCu (x up to 6% weight) alloys have been investigated because of the corrosion and antimicrobial resistance of the Cu containing alloys. Ceramic-reinforced unalloyed titanium based matrix composites have been proposed as alternatives to improve scaffolds performance. However, AM of titanium alloy-based matrix reinforced composites is challenging because the reinforcement materials have different melting temperatures. Nevertheless, AM of titanium-based reinforced composites has a great potential to develop of improved biocompatible metallic scaffolds [208].

Design tools and data management are some of the challenges that need to be addressed in order to fully capitalize on the potential of AM. Improving process control, developing standards and education represent other challenges. In general, the fundamental challenge to obtain functional products via AM is to consider the four M’s (4Ms), namely market, making, materials, and metrology, within a holistic Celtic context [209]. For example, AM printed parts exhibit anisotropic behavior resulting from the nature of AM printing (layer-by-layer) [208].

The big challenge of AM scaffolds is to replicate the in-vivo situation. The healing process is depended on the age. Another issue is vascularization, which causes a lot of materials to fail in-vivo. Tissues need to have a vascular network to grow. In addition, some of the limitations of the AM biomedical applications include the following: [210].

1. Regulatory issues: Regulatory approval is required for any additive manufacturing medical product. Class I devices are considered low-risk, require fewer efforts to be approved, and have been pursued vigorously by the medical industry. However, there are a lot of uncertainties on how AM can affect the safety of class II and class III implants and devices, which are considered high-risk.
2. Limited Materials: [211] Most of the materials used in AM are not biocompatible, and the traditionally used biomaterials cannot be processed with AM techniques. AM technologies need to be improved to process the best available biomaterials.

Two challenges can be identified with the development of AM of titanium-based matrix components. Firstly, powders in a spherical form have limited availabilities while the optimal processing requires spherically shaped powders. Secondly, different melting temperatures exist for the matrix and the reinforcement materials leading to different melting and solidification behaviors [207]. Table 3 lists the general main advantages and limitations of metallic AM scaffolds.

## 11. Future Trends in AM Scaffolds

The Fourth Industrial Revolution, namely Industry 4.0 or Manufacturing 4.0, is directed towards making smart products and building smart factories using intelligent automation technology. Industry 4.0 is built on the following nine technologies: (i) big data and artificial intelligence, (ii) horizontal and vertical integration, (iii) cloud computing, (iv) augmented reality, (v) industrial internet of things, (vi) additive manufacturing (AM), (vii) autonomous robots, (viii) digital twin simulation, and (iv) cybersecurity. AM is thus considered at the forefront of industry 4.0, which focuses on adding values in services and products, and it is expected by 2030 that the global additive manufacturing market will shift from prototyping to mass production of parts [212]. This would require industrialization of additive manufacturing by automating the different processes according to Industry 4.0. AM will thus create new professions and industries [213].

The market of scaffold technology will continue to boom because of the high demand for human tissue repair and regeneration of damaged organs. AM has a great potential in the scaffolds industry because it allows for the development of complex geometries as well as integrating growth factors. In 2019, the global scaffold market size was valued at $969 M, and it is expected to increase at a compound annual growth rate of 9.05% from 2020 to 2027 with a revenue forecast of $1.94 billion in 2027. This market growth is fueled by the development of new biodegradable scaffolds that can mimic the human extracellular matrix. A report published in October 2020 [214] discusses the market size and share and provide a trend analysis on the scaffold technology. From an application point of view, it is reported that using scaffolds for tissue repair is expected to witness the fastest growth from 2020 to 2027 dominating the market with a revenue share of 65.89% in 2019. From a disease point of view, it is reported that the orthopaedics segment had the largest revenue share of 54.17% in 2019 involving 1.6 million bone grafts every year in the USA. It is also reported that North America dominated the scaffold market with a revenue share of 40.73% in 2019. Some major companies in the global scaffold technology include Akron Biotech, where efforts are directed at closely mimicking the architecture of the extracellular matrix in target tissues.

The future trends of AM scaffolds will focus on the following:

1. Manufacturing of patient-specific implants quickly by combining the geometry obtained using CT scans data, design analyses, and AM technologies.
2. New biocompatible implant designs will emerge to allow for cell attachment and growth. Functional composite implants will be developed such as metallic implants coated with ceramic.
3. Meeting the needs for orthopaedic implants by developing AM of low-cost porous titanium composites.
4. Electronic devices will be incorporated with AM printed organs to increase functionality such as the bionic ear to allow for hearing by receiving electromagnetic signals.
5. Improving the cost-effectiveness of AM printed scaffolds.

In the era of Industry 4.0, focus will be on developing new materials for 3D biomedical printing applications. A new field namely, Metal Additive Manufacturing MAM will emerge since metals are the most available common materials. The microstructure of the parts has a great influence on their fatigue properties. Fabrication and printing issues include cost, production speed, improved mechanical properties and surface quality. MAM will need to address these issues in order to meet the industry’s expectations. It is also expected that new processes of AM metals will be improved in the near future to overcome issues such as high unit cost and poor mechanical properties of the printed parts. Research will focus on parameter optimization such as improved powder compounds and precise sintering operations [212].

In order to obtain better surface finish, it is being proposed to improve hybrid processes by combining additive and subtractive manufacturing which allows fabricating complex parts. SLM with precision milling have been proposed to improve surface finish. In the era of the 4th revolution, progress in the hybrid technologies may result from the advancements in information technology.

## 12. Conclusions

This review paper is related to the biomechanics of AM manufactured metallic scaffolds. The different conventional methods to fabricate scaffolds were briefly discussed, which was followed by a presentation of the characteristics of AM metals. A critical discussion has been presented on the factors affecting the scaffold’s technology that include the design of the scaffold, the material used to build the scaffold, and the fabrication process. Ti6Al4V has been identified as an ideal candidate for AM metallic scaffolds. A brief discussion was thus presented on the in-vitro and in-vivo biomechanical studies, and post treatment of Ti6AL4V scaffolds. That was followed by a review of the ongoing effort to develop predictive tools to derive the relationships between structure, processing, properties, and performance of powder-bed additive manufacturing of metals. Finally, the advantages, limitations, and future trends in AM scaffolds are discussed.

## Figures and Tables

**Table 1:** Comparison between different conventional methods for scaffold fabrication (this table is extracted from reference [11]).

Conventional Fabrication Techniques	Advantages	Disadvantages
Freeze-drying	Use in a variety of purposes Capability of obviating high temperatures The pore size is manageable to be controlled by changing the freezing method	High energy consumption Long-term timescale The use of cytotoxic solvents The generation of small and irregular pore sizes (usually in the range of 15 to 35 µm)
Solvent casting and particulate leaching	Fits thin membranes of thin wall three-dimensional specimens High porosity (50–90%) Low cost technique	Time consuming since thin membranes are only used The widespread use of very toxic solvents
Gas foaming	1. Porosity up to 85%	1. If the fabrication process did not change, the product obtained might have a closed pore structure or a solid polymeric skin
Electrospinning	Essential technique for developing nanofibrous scaffolds for the TE Homogeneous mixture made of fibers with high tensile strength	Used solvents can be toxic Problematic to obtain 3D structures as well as sufficient size of pores needed for biomedical applications Process depends on many variables
Thermal-induced phase separation	Construction of the thermoplastic crystalline polymer scaffold Low temperature can be utilized for the integration of bioactive molecules The porosity of fibers is more than 98% a higher surface-to-volume ratio than those constructed	1. Only used for thermoplastic

**Table 2:** A comparison between the seven categories of AM by ASTM (this table is extracted from reference [65]).

CATEGORIES	TECHNOLOGIES	PRINTED “INK”	POWER SOURCE	STRENGTHS/DOWNSIDES
Material Extrusion	Fused Deposition Modeling (FDM)	Thermoplastics, Ceramic slurries, Metal pastes	Thermal Energy	Inexpensive extrusion machine Multi-material printing Limited part resolution Poor surface finish
	Contour Crafting			
Powder Bed Fusion	Selective Laser Sintering (SLS)	Polyamides/Polymer	High-power Laser Beam	High accuracy and details Fully dense parts High specific strength and stiffness Powder handling and recycling Support and anchor structure
	Direct Metal Laser Sintering (DMLS)	Atomized metal powder (17-4 PH stainless steel, cobalt chromium, titanium Ti6Al-4V), Ceramic powder		
	Selective Laser Melting (SLM)			
	Electron Beam Melting (EBM)		Electron Beam	
Vat Photopolymerization	Stereolithography (SLA)	Photopolymer, Ceramics (Alumina, zirconia, PZT)	Ultraviolet Laser	High building speed Good part resolution Overcuring, scanned line shape High cost for supplies and materials
Material Jetting	Polyjet/Inkjet Printing	Photopolymer, Wax	Thermal Energy/Photocuring	Multi-material printing High surface finish Low-strength material
Binder Jetting	Indirect Inkjet Printing (Binder 3DP)	Polymer Powder (Plaster, Resin, Ceramic powder, Metal powder)	Thermal Energy	Full-color objects printing Require infiltration during post-processing Wide material selection High porosities on finished parts
Sheet Lamination	Laminated Object Manufacturing (LOM)	Plastic Film, Metallic Sheet, Ceramic Tape	Laser Beam	High surface finish Low material, machine, process cost Decubing issues
Direct Energy Deposition	Laser Engineered Net Shaping (LENS) Electronic Beam Welding (EBW)	Molten metal powder	Laser Beam	Repair of damage/worn parts Functionally graded material printing Require post-processing machine

**Table 3:** Main advantages and limitations of metallic AM scaffolds.

Advantages	Limitations
AM scaffolds can have complex shapes and can have lightweight cellular structures Patient specific scaffolds Efficient material consumption (minimal waste) thus cost-efficient Mechanical properties can mimic the native structure because the density and topology can be controlled	Concern about health issues due to the release of toxic metal ions Regulatory approval is required for any AM medical product which could be time consuming resulting in a slow developing process Most of the materials used in AM are not biocompatible Increased costs associated with a second surgery for metallic scaffold removal

## Data Availability

Not applicable.
